# Novel metabolic biomarker for early detection and diagnosis to the patients with gastric cardia adenocarcinoma

**DOI:** 10.1002/cam4.7015

**Published:** 2024-03-16

**Authors:** Meng Xia Wei, Zheng Yang, Pan Pan Wang, Xue Ke Zhao, Xin Song, Rui Hua Xu, Jing Feng Hu, Kan Zhong, Ling Ling Lei, Wen Li Han, Miao Miao Yang, Fu You Zhou, Xue Na Han, Zong Min Fan, Jia Li, Ran Wang, Bei Li, Li Dong Wang

**Affiliations:** ^1^ State Key Laboratory of Esophageal Cancer Prevention & Treatment and Henan Key Laboratory for Esophageal Cancer Research of The First Affiliated Hospital of Zhengzhou University Zhengzhou University Zhengzhou Henan Province PR China; ^2^ School of Life Science Zhengzhou University Zhengzhou Henan Province PR China; ^3^ Department of Thoracic Surgery Anyang Tumor Hospital Anyang Henan Province PR China; ^4^ Department of Language Zhengzhou White Gown Translation Co., Ltd. Zhengzhou PR China

**Keywords:** bile acid, gastric cardia adenocarcinoma, HER2, metabolomics, stage

## Abstract

**Background:**

Gastric cardia adenocarcinoma (GCA) is classified as Siewert type II adenocarcinoma at the esophagogastric junction in Western countries. The majority of GCA patients do not exhibit early warning symptoms, leading to over 90% of diagnoses at an advanced stage, resulting in a grim prognosis, with less than a 20% 5‐year survival rate.

**Method:**

Metabolic features of 276 GCA and 588 healthy controls were characterized through a widely‐targeted metabolomics by UPLC‐MS/MS analysis. This study encompasses a joint pathway analysis utilizing identified metabolites, survival analysis in both early and advanced stages, as well as high and negative and low expression of HER2 immunohistochemistry staining. Machine learning techniques and Cox regression models were employed to construct a diagnostic panel.

**Results:**

A total of 25 differential metabolites were consistently identified in both discovery and validation sets based on criteria of *p* < 0.05, (VIP) ≥ 1, and FC ≥ 2 or FC ≤ 0.5. Early‐stage GCA patients exhibited a more favorable prognosis compared to those in advanced stages. HER2 overexpression was associated with a more positive outcome compared to the negative and low expression groups. Metabolite panel demonstrated a robust diagnostic performance with AUC of 0.869 in discovery set and 0.900 in validation set.

**Conclusions:**

A total of 25 common and stable differential metabolites may hold promise as liquid non‐invasive indicators for GCA diagnosis. HER2 may function as a tumor suppressor gene in GCA, as its overexpression is associated with improved survival. The downregulation of bile acid metabolism in GCA may offer valuable theoretical insights and innovative approaches for precision‐targeted treatments in GCA patients.

## 1 INTRODUCTION

Gastric cardia adenocarcinoma (GCA) is a prevalent malignancy of the digestive tract in China, primarily manifesting in the gastric cardia region, situated within a 2 cm radius of the esophagogastric junction.^1^ This particular cancer, now classified as Siewert type II adenocarcinoma at the esophagogastric junction in Western medical nomenclature, ranks among the most frequently encountered malignancies in the country. Over recent decades, there has been a noteworthy surge in the incidence, morbidity, and mortality rates of GCA.^2,^, ^3^, ^4^ Clinical diagnoses typically occur when the disease has advanced to a late stage, primarily due to its insidious nature and the absence of overt symptoms that could facilitate early detection and intervention in precancerous lesions.^5^ Endoscopic examination coupled with biopsy stands as the gold standard for screening GCA.^6^ Early detection through screening plays a pivotal role in identifying individuals with precancerous cardia mucosal lesions in high‐risk regions of China.^7^ However, the cost and invasiveness of endoscopic screening make it less feasible for large‐scale population‐based initiatives. Non‐invasive biomarkers, in contrast, offer a more cost‐effective and practical approach to population‐wide screening. In this population‐based study, our primary objective is to pinpoint potential metabolic biomarkers for the early screening and detection of GCA, while also establishing an optimal model for early GCA screening. Early diagnosis, prevention, and treatment represent critical strategies for curbing the incidence and mortality of GCA, underscoring the significance of identifying non‐invasive serum‐based early detection indicators.

Metabolomics, following genomics and transcriptomics, stands as a pivotal component of systems biology, with its inception traced back to the pioneering work of Nicholson, Lindon, and Holmes in 1999.^8^, ^9^ This emerging field focuses on the comprehensive qualitative and quantitative analysis of all low molecular weight metabolites (typically below 1000 Da) within a specific organism or cellular context during defined physiological and pathological states. Metabolomics research centers on the profiling of endogenous small‐molecule metabolites in the human body, offering insights into systemic metabolic alterations through high‐throughput analytical techniques. Metabolomic alterations occur in response to changes in physiological, pathological, and other factors within the body. Metabolomics research offers a comprehensive view of how these diverse factors impact the organism, providing a holistic representation of their effects.^10^ The uniqueness of metabolomics is its real‐time capacity to capture ongoing bodily changes, rendering it a potent tool for unveiling dynamic shifts closely linked to phenotype.^11^ Even subtle genetic and protein variations can be magnified at the metabolic level. By discerning differences in substance types and concentrations, potential disease course‐related biomarkers can be identified. Over recent decades, metabolomics has evolved into a pivotal field, delving into metabolic processes and unearthing potential biomarkers, thus emerging as a potent instrument for scrutinizing metabolic perturbations across a spectrum of diseases.^12^, ^13^, ^14^, ^15^ Biomarker discovery assumes a pivotal role in gauging disease status,^16^ monitoring drug sensitivity,^17^ and assessing physiological conditions.^18^ The aberrant metabolism characteristic of cancer has gained significant recognition, shedding light on tumor pathogenesis and unveiling prospective clinical targets for therapeutic interventions.

In this study, we conducted a comprehensive analysis of the metabolic profiles of GCA patients and healthy controls. We employed a nontargeted metabolomics profiling approach using ultra‐high performance liquid chromatography‐mass spectrometry/mass spectrometry (UPLC‐MS/MS). The study comprised 864 subjects, including GCA patients and healthy controls, randomly allocated into discovery and validation sets. Our analyses encompassed differential metabolite profiling, survival analysis, visualization of metabolite disparities between GCA patients and healthy controls, discrimination between early and advanced stage GCA patients, stratification based on HER2 immunohistochemistry staining, and pathway analysis using the KEGG database. We utilized machine learning techniques and Cox regression models to establish a diagnostic metabolite panel.

## 2 MATERIALS AND METHODS

### 2.1 Study participants

This study involved a cohort of 864 participants, consisting of 276 GCA patients (the experimental group) and 588 non‐GCA individuals (the control group). GCA patients in the experimental group were diagnosed between 1987 and 2020 and were followed up until 2023. The study cohort was randomly partitioned into a training set, comprising 138 GCA participants and 363 controls, as well as a validation set, consisting of 138 GCA participants and 225 controls. Comprehensive clinical characteristics were analyzed, including gender, age, geographic incidence, smoking, alcohol consumption, family history, differentiation, lymph node involvement (positive/negative), TNM stage according to the 6th UICC classification, disease staging (early/advanced), and variations in HER2 expression.

The GCA participants were selected based on their absence of prior chemoradiotherapy, while healthy participants were free from any history of esophageal or cardia‐related diseases. A majority of the GCA individuals in the discovery set were sourced from the Anyang Cancer Hospital, with the remaining recruited from various regional hospitals within Henan. The validation cohort of 138 GCA patients comprised individuals from Anyang Cancer Hospital, Linzhou People's Hospital, and Linzhou Cancer Hospital. All participants provided written informed consent, and the study received approval from the ethics committees of each respective cohort. The diagnosis was confirmed through histopathological examination.

Inclusion criteria for GCA participants were as follows: (1) Definitive histopathological diagnosis of the specified pathological type; (2) Pathological confirmation of cardia adenocarcinoma; (3) Absence of prior exposure to chemotherapy, radiotherapy, or other antineoplastic therapies; (4) Absence of concomitant malignancies; (5) Absence of other metabolic disorders; (6) Absence of concurrent co‐infections, as evidenced by the absence of symptoms such as fever and hematological abnormalities.

Inclusion criteria for healthy controls involved individuals who had undergone upper gastroscopic biopsy exclusion and were free of malignant tumors or other metabolic diseases during the period spanning from 2012 to 2020. These participants were randomly selected and evenly distributed between the discovery and verification sets.

All GCA patients received independent confirmation of their diagnosis as gastric cardia adenocarcinoma from two pathologists. A positive family history was established if the proband had one or more cancer‐affected relatives across three consecutive generations. Regions with an esophageal cancer incidence exceeding 60 cases per 10 million were categorized as high incidence areas, while others were considered low incidence areas. Early cancer included stages I and IIA, and advanced cancer consisted of stages IIB, III, and IV. Ongoing survival follow‐up involved telephone or home inquiries every 3–6 months. Each participant provided informed consent, and ethical approval was granted by the Medical Ethics Committee of the First Affiliated Hospital of Zhengzhou University.

### 2.2 Metabolomics preparation and analysis

#### 2.2.1 Blood sample

Fasting blood samples were obtained from GCA patients during morning hours prior to surgery, while healthy control participants provided blood samples during site investigations or gastrointestinal endoscopy. These blood samples were collected into centrifuge tubes and allowed to stand at either 37°C or room temperature for 1 h to stratify components. Subsequently, samples were centrifuged at 3000 rpm (with a radius of 8 cm) at room temperature for 10 min, and the resulting supernatant was transferred to clean centrifuge tubes. Further centrifugation was conducted at 12,000 rpm (with a radius of 8 cm) at 4°C for 10 min, with the supernatant being transferred into 1.5 mL centrifuge tubes, each containing 0.2 mL of the sample. The samples were then stored at −80°C and transported in dry ice.

The blood samples from both GCA patients and healthy controls were processed under ice‐cold conditions as part of the follow‐up protocol. Initially, after being removed from the −80°C freezer, the samples were vortexed for 10 s to ensure thorough mixing and dissolution. Subsequently, 300 μL of chromatographic‐grade methanol was added to 50 μL of the blood sample within numbered centrifuge tubes. This mixture was then vortexed for 3 min to facilitate metabolite dissolution. The resulting extraction blood samples were subjected to centrifugation for 10 min at 12,000 rpm. Following this step, 200 μL of the supernatant was transferred to another numbered centrifuge tube and centrifuged for 3 min at 12,000 rpm after being stored at −20°C for 30 min at 4°C. Finally, 150 μL of the supernatant was subjected to analysis using UPLC‐MS.

#### 2.2.2 UPLC‐MS/MS condition

The serum metabolites of both GCA and healthy control participants were investigated using a metabolomics approach employing ultra‐performance liquid chromatography‐mass spectrometry (UPLC‐MS/MS) utilizing the QTRAP® system (https://sciex.com/, SCIEX, USA). The UPLC‐MS methodology encompassed two key sections: Section 1 pertaining to HPLC conditions and Section 2 covering ESI‐QTRAP‐MS/MS.

Section 1: The mobile phase A consisted of ultra‐pure water with 0.1% chromatographic formic acid, while mobile phase B comprised chromatographic‐grade acetonitrile with 0.1% chromatographic formic acid. Of the previously mentioned 15 μL supernatant, 2 μL was injected into the detector and subjected to separation on a Water ACQUITY UPLC HSS T3 C18 chromatographic column (1.8 μm, 2.1 mm × 100 mm) maintained at 40°C, with a flow rate of 0.4 mL/min. Elution for phase A started at 95%, decreased to 10% over 10 min, then returned to 95% in 11.1 min and was maintained for 3 min.

Section 2: Analysis was performed in both positive and negative ion modes. For the ESI source, parameters included a capillary voltage of −4500 V (negative) and 5500 V (positive), a capillary temperature of 500 °C, sheath and auxiliary gas flow (N2) at 55 psi (GS I) and 60 psi (GS II), and a sweep gas at 25 psi. Instrument tuning and mass calibration were conducted with 10 and 100 μmol/L polypropylene glycol solutions in QQQ and LIT modes, respectively. A specific set of MRM transitions was monitored for each period corresponding to the elution of metabolites during that period.

Instrument control, data acquisition, and data analysis were executed using Xcalibur software version 2.1.

### 2.3 Analysis method

#### 2.3.1 Data processing

Following mass spectrometry data analysis with Analyst 1.6.3 software (AB SCIEX, Ontario, Canada), metabolite qualitative analysis was performed based on criteria such as retention time, ion pair, and secondary spectra, utilizing the MetWare database (http://www.metware.cn/) and publicly accessible metabolite repositories, including HMDB (http://hmdb.ca/) and MassBank (http://massbank.jp/). Quantitative analysis comprised the following steps: characteristic ions for each substance were screened using a triple quadrupole instrument, and the signal intensities of these ions were obtained. Subsequently, chromatographic peaks were integrated and corrected using the MultiQuant software to determine the relative content of the respective substances represented by peak areas. Metabolite annotation was conducted with reference to the KEGG compound database (http://www.kegg.jp/kegg/compound).

#### 2.3.2 Quality control

Data quality assessment involved principal component analysis (PCA) using the princomp function in R and calculation of the coefficient of variation (CV) as the ratio of standard deviation to mean, performed with Microsoft Excel 2016 and the ggplot2 package in R. Unsupervised PCA was executed using the prcomp function in R after unit variance scaling. Pattern recognition analysis was conducted with the software package Simca version 14.1 (Umetrics AB, Umeå, Sweden). Response variables were centered and scaled to Pareto variance, with base weights computed as one divided by the square root of the standard deviation of the response variables. In order to normalize skewed distributions, log transformations were applied for non‐linear data conversions. To mitigate inter‐subject variability and identify significant contributors among endogenous metabolites for classification, orthogonal partial least squares‐discriminant analysis (OPLS‐DA) was performed.^19^, ^20^

#### 2.3.3 Statistical analyses

Multivariate statistical analyses included PCA and Orthogonal Partial Least Squares‐Discriminant Analysis (OPLS‐DA) conducted using the “ropls” package in R. The model quality of OPLS‐DA was assessed using R2Y and Q2 values. Volcano plots were constructed based on log2 fold changes and ‐log10 (*p*‐value) using R. Heatmaps were generated and visualized using the pheatmap package. Statistically significant metabolites were identified based on a threshold of *p* < 0.05 (using Student's *t*‐test or Wilcoxon test), and the variable importance in projection (VIP) scores derived from the OPLS‐DA model were utilized as supplementary information.

For machine learning and Cox regression, the analysis followed a systematic approach: First, machine learning analyses were conducted independently on the two sets, followed by a comparison of metabolites consistent between both sets. The data preprocessing consisted of the following steps: (1) Data inspection and formatting, check data and group file samples, and determine control/case groups; (2) Statistics on raw data; (3) Split the discovery set and validation set according to 7:3; second, biomarker screening was performed within the discovery set, following these steps: (1) Inclusion criteria of FC < 0.67 or FC > 1.5, and *p* < 0.05 were applied; (2) Selection the intersection of the two inclusion criteria, and then calculation the correlation, when the correlation value >0.9, only one of them will remain; (3) Screening by Lasso, total 15 rounds of random calculations were taken, and then remaining the variables with an average variable importance <15; (4) Important score of multi‐method for Lasso analysis results, and take the top 10 scores as the panel; (5) Stepwise screening followed by Lasso result. Note that there are two panels used in subsequent modeling: Panel1: 10 metabolite panels with the top 10 variable scores; Panel2: Metabolite panel obtained stepwise; third, modeling in discovery set. Three methods of LR/SVM/RF were used to model the two different panels mentioned above by discovery set data. Fourth, validation in validation set.

Kaplan–Meier survival curves were generated to calculate and compare survival rates between groups, with log‐rank tests applied for comparison (utilizing the survival and survminer packages). The Cox proportional hazards regression analysis was conducted to evaluate prognostic factors for overall survival using the survival package. Additionally, pathway analysis was carried out using the KEGG pathway database through the MetaboAnalyst 5.0 online software.

Statistical analyses were performed using R software (version 4.0.4), with statistical significance defined as *p* < 0.05.

## 3 RESULTS

### 3.1 Demographic characteristics of the study population

**FIGURE 1:**
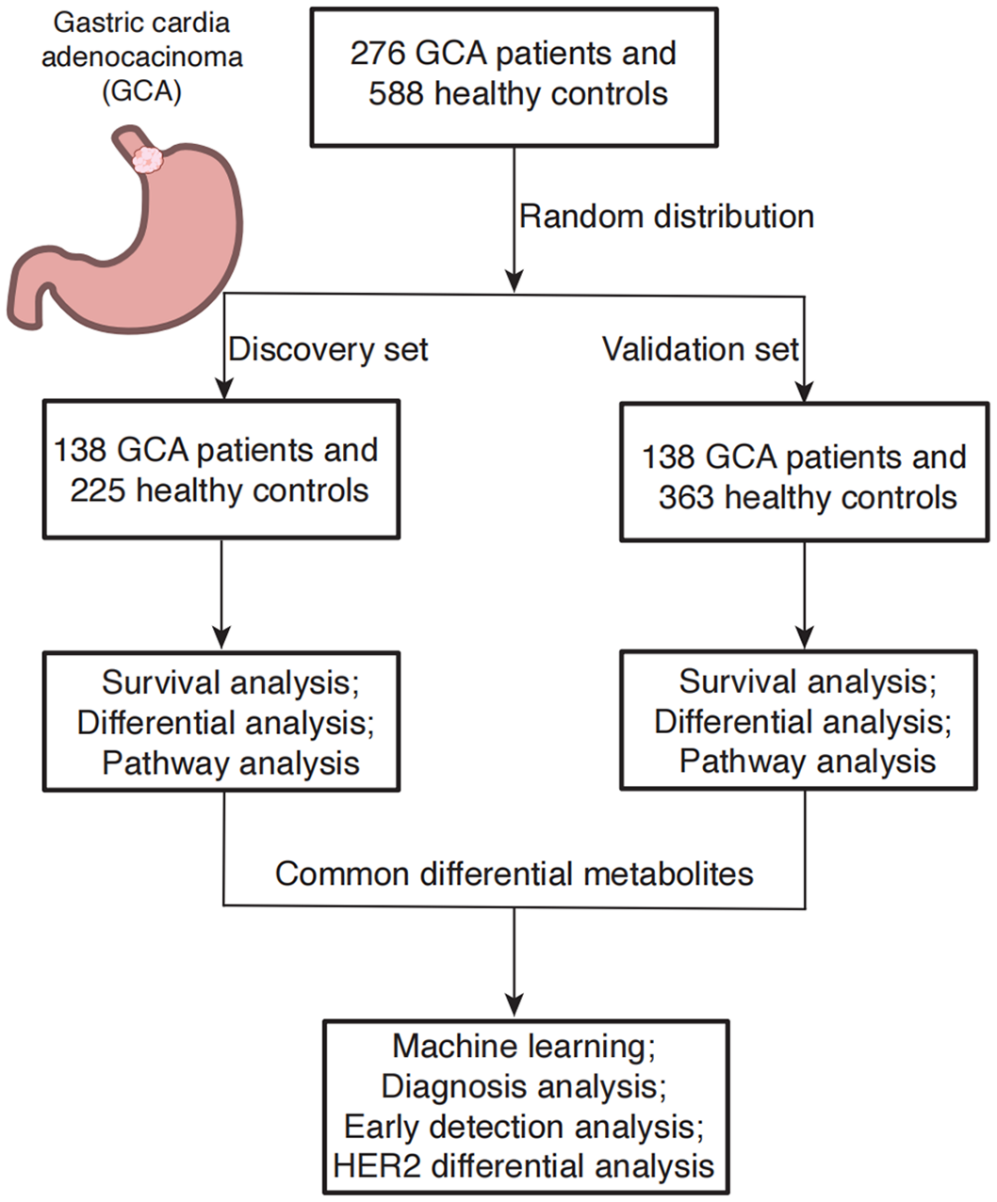
Study design. There are a total of 864 participants, including 588 healthy volunteers, 276 GCA patients in the discovery (138 GCA patients and 363 healthy control) and validation sets (138 GCA patients and 225 healthy control).

A total of 864 participants were enrolled in this study, comprising 588 healthy volunteers and 276 patients diagnosed with GCA from two independent cohorts, as depicted in Figure 1. The study was divided into a discovery set, which consisted of 363 healthy volunteers and 138 GCA patients, and a validation set, comprising 225 healthy volunteers and 138 GCA patients. Key clinical characteristics, such as gender, age, family history, geographical incidence, smoking and drinking habits, differentiation, lymph node metastasis, and TNM stage, were comprehensively summarized for both GCA patients and healthy control subjects in Table 1.

**TABLE 1:** Demographic characteristics of the study population, *n* (%).

Characteristics	Overall GCA	Overall healthy control	Discovery group (*n* = 501)		Validation group (*n* = 363)	
			GCA	Healthy control	GCA	Healthy control
	276 (100.00)	588 (100.00)	138 (50.00)	363 (61.73)	138 (50.00)	225 (38.27)
Gender (%)						
Male	220 (79.71)	250 (42.52)	111 (80.43)	142 (39.11)	109 (78.99)	108 (48.00)
Female	56 (20.29)	338 (57.48)	27 (19.57)	221 (60.88)	29 (21.01)	117 (52.00)
Age (mean ± (SD))	62.78 ± 7.22	60.58 ± 9.92	64.66 ± 7.15	63.53 ± 9.06	60.91 ± 6.82	55.75 ± 9.37
High/low incidence area (%)						
High	202 (73.19)	588 (100.00)	85 (61.59)	363 (100.0)	117 (84.78)	225 (100.00)
Low	74 (26.81)	0 (0.00)	53 (38.41)	0 (0.00)	21 (15.22)	0 (0.00)
Smoking (%)						
No	143 (51.81)	–	74 (53.62)	–	69 (50.00)	–
Yes	129 (46.74)	–	64 (46.38)	–	65 (47.10)	–
Missing	4 (1.45)	–	–	–	4 (2.90)	–
Drinking (%)						
No	165 (59.78)	–	98 (71.01)	–	67 (48.55)	–
Yes	106(38.41)	–	40 (28.99)	–	66 (47.83)	–
Missing	5(1.81)	–	–	–	5 (3.62)	–
Family history (%)						
Negative	111 (40.22)	–	34 (24.64)	–	77 (50.73)	–
Positive	159 (57.61)	–	104 (75.36)	–	55 (39.86)	–
Missing	6 (2.17)	–	–	–	6 (4.35)	–
Differentiation (%)						
Well	6 (2.17)	–	3 (2.17)	–	3 (2.17)	–
Moderately	129 (46.74)	–	51 (36.96)	–	78 (56.52)	–
Poorly	127 (46.01)	–	74 (53.62)	–	53 (38.41)	–
Missing	14 (5.07)	–	10 (7.25)	–	4 (2.90)	–
Lymph node metastasis (%)						
Negative	85 (30.80)	–	50 (36.23)	–	35 (25.36)	–
Positive	191 (69.20)	–	88 (63.77)	–	103 (74.64)	–
TNM stage (%)						
I	12 (4.35)	–	12 (8.70)	–	0 (0.00)	–
II	82 (29.71)	–	44 (31.88)	–	38 (27.54)	–
III	162 (58.70)	–	63 (45.65)	–	99 (71.74)	–
IV	19 (6.88)	–	19 (13.77)	–	0 (0.00)	–
Missing	1 (0.36)	–	0 (0.00)	–	1 (0.73)	–
Early/middle‐advanced stage (%)						
Early stage	81 (27.35)	–	49 (35.51)	–	32 (23.19)	–
Advanced stage	194 (70.29)	–	89 (64.49)	–	105 (76.09)	–
Misusing	1 (0.36)	–	0 (0)	–	1 (0.01)	–
HER2 expression (%)						
High	15 (5.43)	–	9 (6.52)	–	6 (4.35)	–
Negative and low	207 (75)	–	122 (88.41)	–	85 (61.59)	–
2+/missing	54 (19.57)	–	7 (5.07)	–	47 (34.06)	–

*Note*: High/Low incidence area: The areas with a mortality rate of more than 60/100,000 are high incidence areas, and the mortality rate low than 60/100,000 are low incidence areas.

### 3.2 Quality control and differential metabolites analysis

**FIGURE 2:**
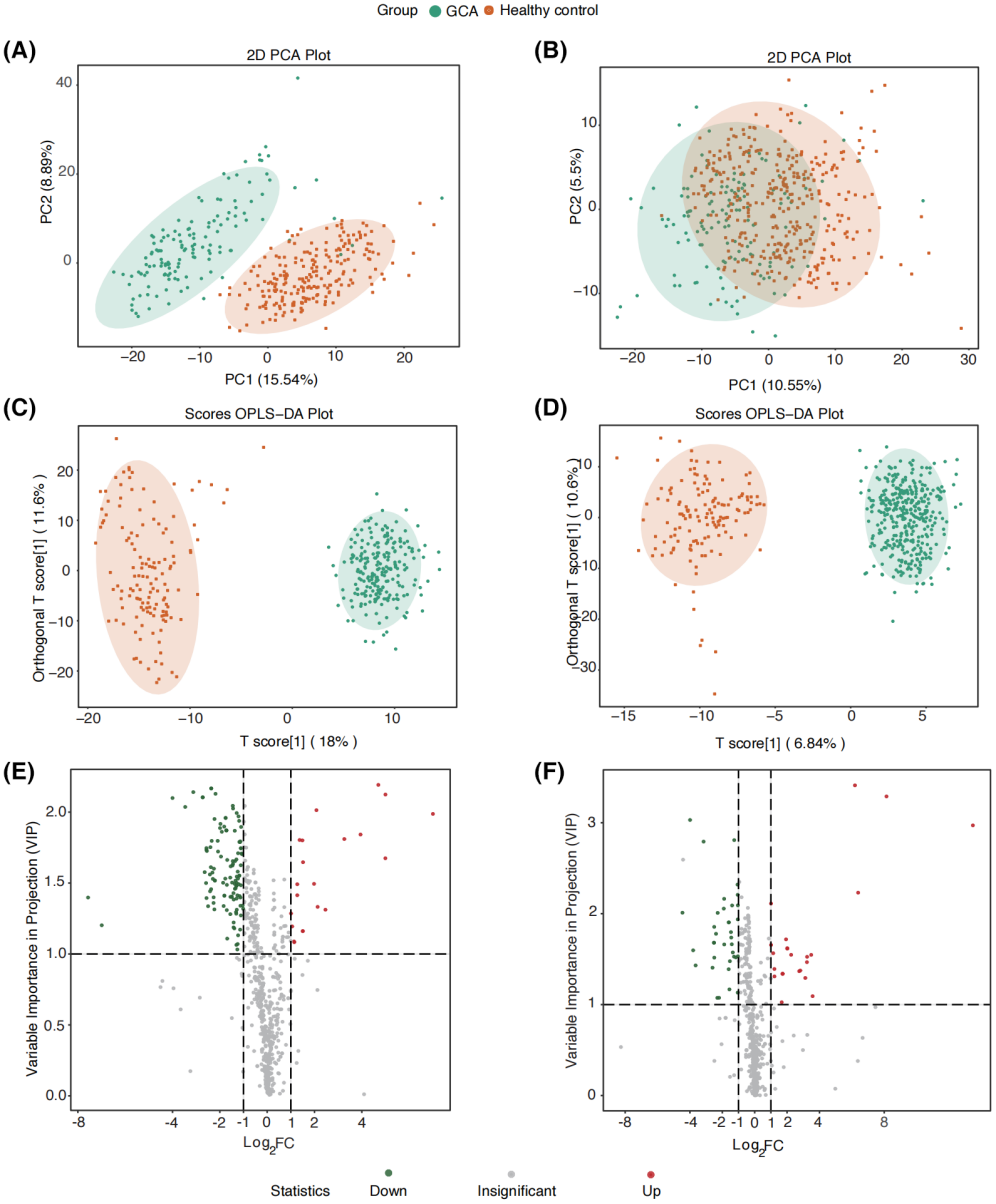
Principal component analysis (PCA) of discovery set (A) and validation set (B); orthogonal partial least squares discriminant analysis (OPLS‐DA) analysis of discovery set (C) and validation set (D); and volcano plot of differential metabolites for discovery set (E) and validation set (F).

PCA revealed distinct differences between samples obtained from healthy controls and GCA participants, as illustrated in Figure 2A,B. Notably, no significant outliers were detected, underscoring the robustness of the data analysis.^21^ The most effective discrimination between GCA and healthy control groups was achieved through the principal components within the discovery set, accounting for 15.54% and 8.89% of the total dataset variance, respectively. In the validation set, a similar clear demarcation between GCA patients and healthy controls was observed, affirming the comparability of the two sets despite their independent execution.

Subsequently, orthogonal partial least squares discriminant analysis (OPLS‐DA) revealed substantial distinctions between healthy controls and GCA participants (as depicted in Figure 2C for the discovery set and Figure 2D for the validation set). Notably, GCA participants were predominantly clustered in the negative quadrant, while healthy controls were concentrated in the positive quadrant, signifying pronounced dissimilarities between the two groups in both sets. The model's effectiveness was underscored by significant fitting parameters, with R2Y (0.961) and Q2 (0.953) in the discovery set. R2Y represents the proportion of explainable variation to total variation within the OPLS‐DA model, and Q2 signifies the ratio of predictable variation to total variation, indicating the model's explanatory power. In conclusion, a marked metabolic disparity was observed between GCA patients and healthy controls, with the OPLS‐DA model demonstrating strong fitness and predictive capability.

**FIGURE 3:**
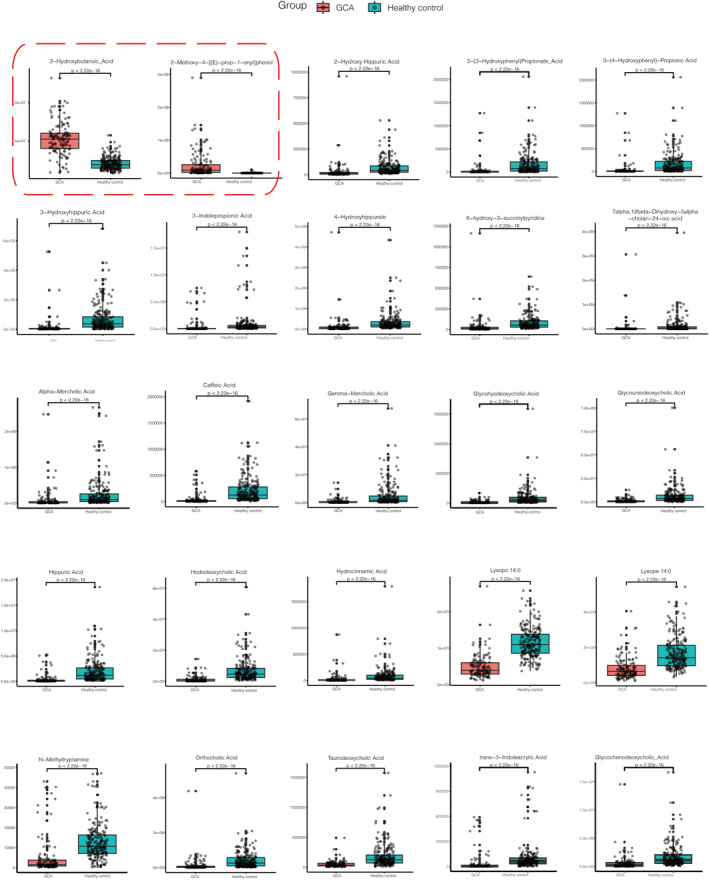
Boxplots for 25 differential metabolites for discovery set with the criterion of *p* < 0.05, VIP ≥ 1, and FC ≥ 2 or FC ≤ 0.5. The two boxplots with red frame were upregulated metabolites 2‐hydroxybutanoic acid and 2‐methoxy‐4‐[(E)‐prop‐1‐enyl]phenol. And the rest were downregulated metabolites hododeoxycholic acid, glycoursodeoxycholic acid, glycochenodeoxycholic acid, caffeic acid, hippuric acid, hydrocinnamic acid, lysope 14:0, 3‐(3‐hydroxyphenyl)propionate acid, 3‐hydroxyhippuric acid, 2‐hydroxy hippuric acid, 3‐(4‐hydroxyphenyl)‐propionic acid, taurodeoxycholic acid, alpha‐mercholic acid, gamma‐mercholic acid, orthocholic acid, 6‐hydroxy‐3‐succinylpyridine, 7alpha,12beta‐dihydroxy‐5alpha‐cholan‐24‐oic acid, glycohyodeoxycholic acid, N‐methyltryptamine, 3‐indolepropionic acid, lysopc 14:0, trans‐3‐indoleacrylic acid, and 4‐hydroxyhippurate.

**FIGURE 4:**
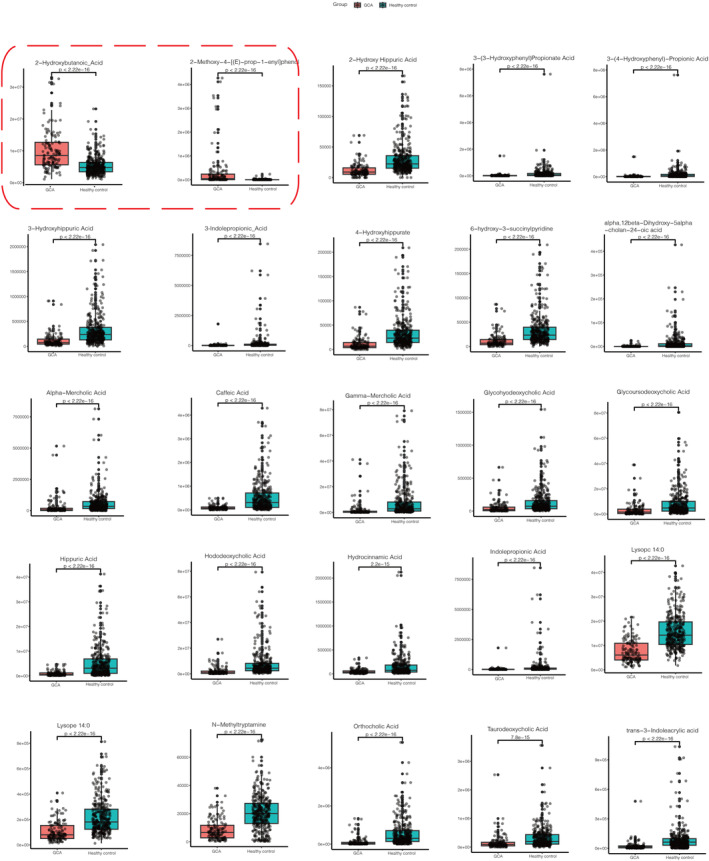
Boxplots for 25 differential metabolites for validation set with the criterion of *p* < 0.05, VIP ≥ 1, and FC ≥ 2 or FC ≤ 0.5. The two boxplots with red frame were upregulated metabolites 2‐hydroxybutanoic acid and 2‐methoxy‐4‐[(E)‐prop‐1‐enyl]phenol. And the rest were downregulated metabolites hododeoxycholic acid, glycoursodeoxycholic acid, glycochenodeoxycholic acid, caffeic acid, hippuric acid, hydrocinnamic acid, lysope 14:0, 3‐(3‐hydroxyphenyl)propionate acid, 3‐hydroxyhippuric acid, 2‐hydroxy hippuric acid, 3‐(4‐hydroxyphenyl)‐propionic acid, taurodeoxycholic acid, alpha‐mercholic acid, gamma‐mercholic acid, orthocholic acid, 6‐hydroxy‐3‐succinylpyridine, 7alpha,12beta‐dihydroxy‐5alpha‐cholan‐24‐oic acid, glycohyodeoxycholic acid, N‐methyltryptamine, 3‐indolepropionic acid, lysopc 14:0, trans‐3‐Indoleacrylic acid, and 4‐hydroxyhippurate.

Volcano plot (Figure 2E,F) showed the upregulated and downregulated metabolites for discovery set and validation set, respectively. We totally detected 865 metabolites showed in Table S1, 21 upregulated and 117 downregulated metabolites for discovery set, 25 upregulated and 36 downregulated metabolites for validations with the criterion of *p* < 0.05, VIP ≥ 1, and FC ≥ 2 or FC ≤ 0.5. The detail information for every metabolite is shown in Tables S2 and S3. A total of 25 common metabolites (2 up and 23 downregulated) were detected for both discovery and validation sets, with eight bile acids, six organic acid and its derivatives, three benzene and substituted derivatives, two metabolites for amino acid and its metabolomics; heterocyclic compounds; tryptamines, cholines, pigments; glycerophospholipids. Boxplots of the 25 differential metabolites were showed in Figures 3 and 4, we can observe the two metabolites boxplots with red frame (2‐hydroxybutanoic acid and 2‐methoxy‐4‐[(E)‐prop‐1‐enyl]phenol) were higher enrichment in GCA patients than healthy controls. The others were lower enrichment in GCA patients than healthy controls for metabolites of hododeoxycholic acid, glycoursodeoxycholic acid, glycochenodeoxycholic acid, caffeic acid, hippuric acid, hydrocinnamic acid, lysope 14:0, 3‐(3‐hydroxyphenyl)propionate acid, 3‐hydroxyhippuric acid, 2‐hydroxy hippuric acid, 3‐(4‐hydroxyphenyl)‐propionic acid, taurodeoxycholic acid, alpha‐mercholic acid, gamma‐mercholic acid, orthocholic acid, 6‐hydroxy‐3‐succinylpyridine, 7alpha,12beta‐Dihydroxy‐5alpha‐cholan‐24‐oic acid, glycohyodeoxycholic acid, N‐methyltryptamine, 3‐indolepropionic acid, lysopc 14:0, trans‐3‐indoleacrylic acid, and 4‐hydroxyhippurate.

### 3.3 Survival analysis for clinical characteristics

**FIGURE 5:**
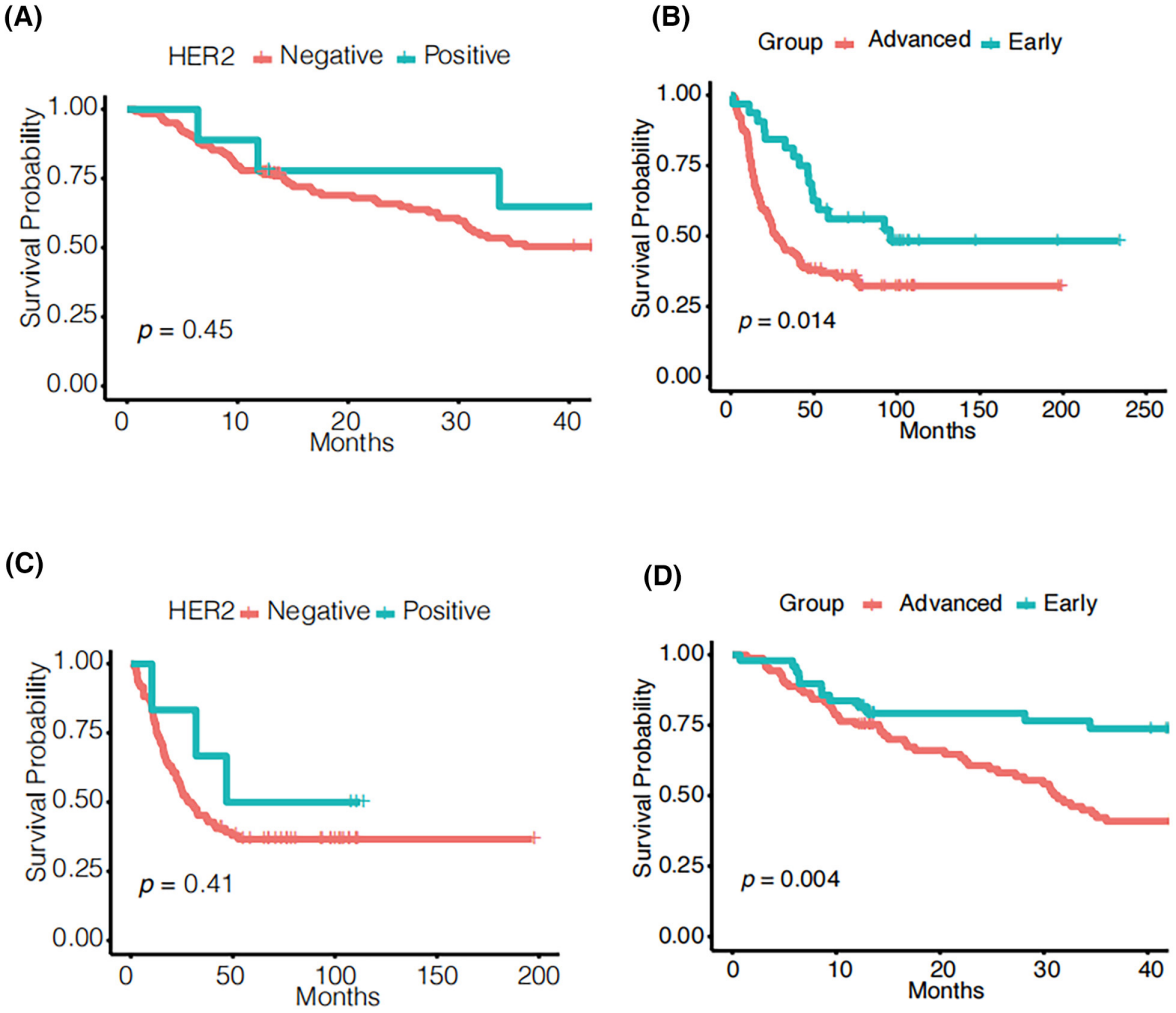
Survival analysis of HER2 expression (A, discovery set; C, validation set) and clinical stage (B, discovery set; D, validation set).

Kaplan–Meier survival analysis for discovery set and validation set were calculated, and the *p*‐value result were showed in Table 2. We observed that the clinical characteristics including to smoking, lymph node, stage, early (I–IA stage)/advanced stage (IIB–IV stage) showed significant difference of overall survival in two patient groups (*p* < 0.05). Additionally, we also observed that the HER2 expression in two groups showed insignificant *p* = 0.45 (discovery set) and *p* = 0.41 (validation set) in immunohistochemistry in positive (3+) better than in negative and low (0 and 1+) in Figure 5A,B, but the two line were separated. Generally, HER2 overexpression confers worse biological behavior and clinical aggressiveness in breast cancer.^22^ We speculated that HER2 overexpression is an important indicator of good prognosis in GCA. We also seen that patients stage variation (early vs. advanced stage) showed varied survival (*p* = 0.004 for discovery set, *p* = 0.014 for validation set). Hence, we compared two groups metabolites difference, including early and advanced stage groups and HER2 negative and low and high expression groups.

**TABLE 2:** Kaplan–Meier survival analysis of clinical characteristics.

	*p*‐value of discovery set	*p*‐value of validation set
Gender	0.0033	0.1
Diagnosis age	0.6	0.46
Incidence area	0.64	0.0039
Urban/rural	0.52	0.66
Smoking	0.0038	0.047
Drinking	0.19	0.023
Family history	0.066	0.86
BMI	0.14	0.58
Blood	0.53	0.78
Differenation	0.35	0.29
Lymph node	0.0024	0.0096
Stage	0.014	0.0032
Early/advanced stage	0.004	0.014
HER2 expression	0.45	0.41

### 3.4 Metabolomic profile of matched GCA and healthy control, early and advanced stage of GCA, and HER2 expression difference

**FIGURE 6:**
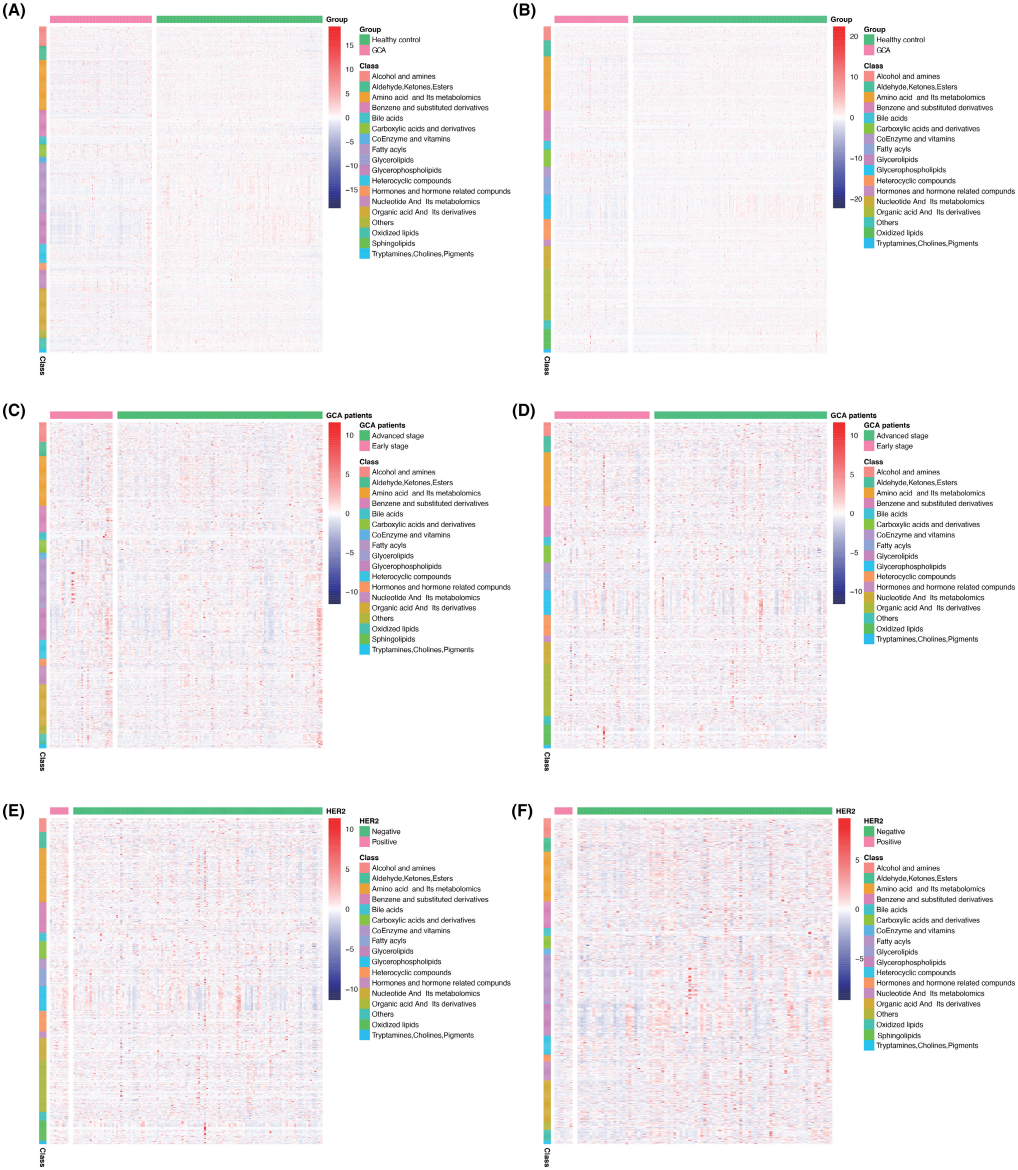
Heatmap summarizing differential metabolites in GCA patients versus healthy controls based on metabolite classes were showed in (A) and (B). Color bars at the right from top to down indicate samples from patients (purple) and controls (green), metabolites classes, and relative abundance of upregulated (red) and downregulated (blue) metabolites, respectively. Each column represents an individual subject, and each row a metabolite; heatmap of GCA patients in early stage and advanced stage in discovery set (C) and validation set (D); HER2 negative and low and high expression groups for GCA patients in discovery set (E) and validation set (F).

Metabolomic data were also visualized using heatmaps, with metabolites arranged by major classes (Figure 6). In summary, the comprehensive shifts in the metabolic profile of GCA patients were markedly distinct when compared to the healthy control group, as demonstrated in Figure 6A,B. Particularly noteworthy were the notable variations observed in metabolites related to bile acids, amino acids and their derivatives, glycerolipids, and glycerophospholipids. Discernible distinctions between early‐stage and advanced stage GCA patients are depicted in Figure 6C,D. Notably, amino acids and their metabolites exhibited upregulation, while glycerophospholipids displayed downregulation in advanced stage GCA patients. Furthermore, differences in HER2 expression were evident in the low and high expression groups, as illustrated in Figure 6E,F. Specifically, amino acids and their metabolites exhibited upregulation in the HER2 overexpression group, whereas glycerophospholipids were downregulated in the HER2‐negative and low‐expression group.

### 3.5 Established the biomarker diagnostic model

**FIGURE 7:**
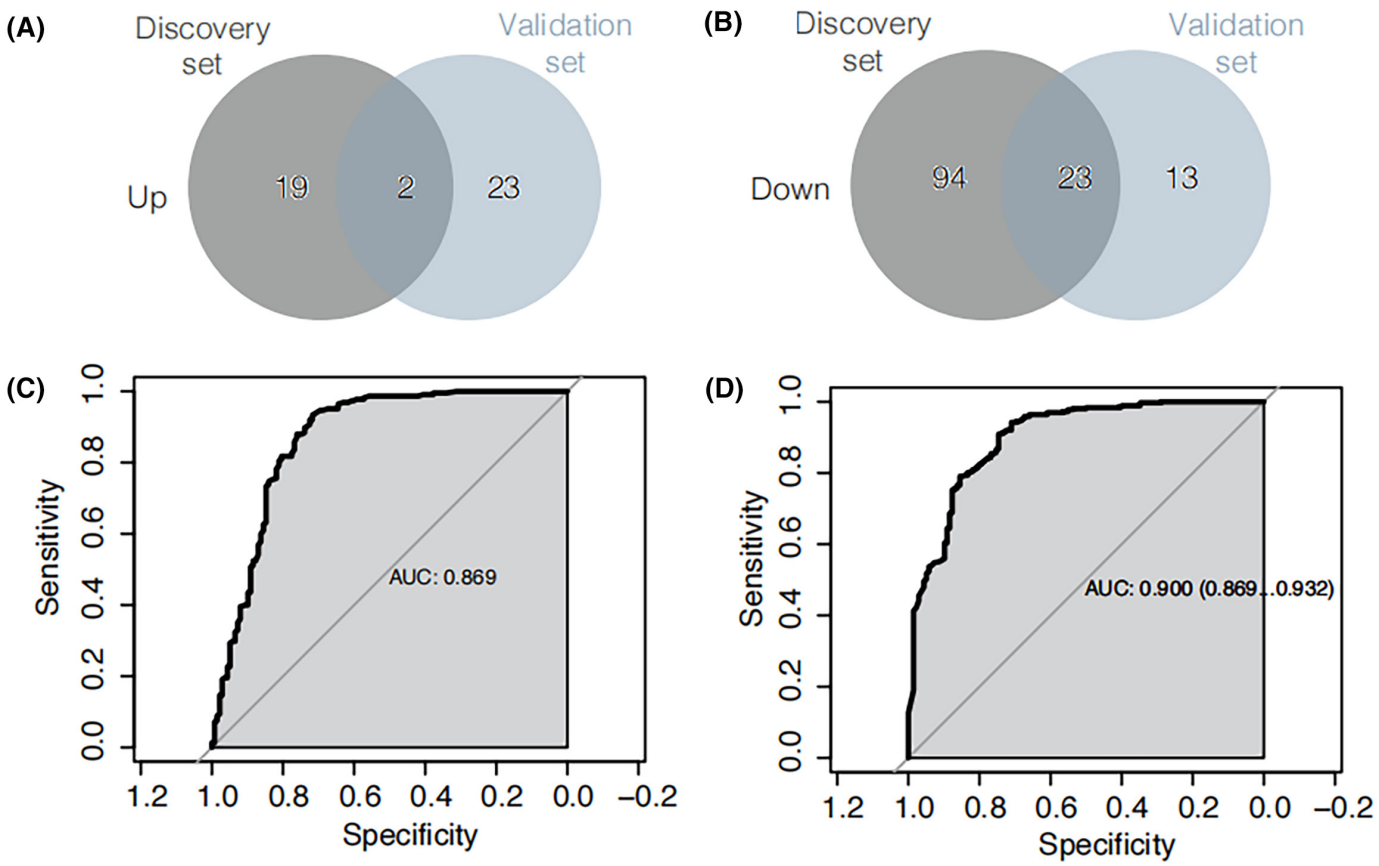
Venn diagram of differential upregulated metabolites overlaps from discovery set and validation set (A). Venn diagram of differential downregulated metabolites overlaps from discovery set and validation set (B). Machine learning and Cox regression model for discovery set (C) and validation set (D).

Differential upregulated and downregulated metabolites overlaps among discovery set and validation set (with the criterion of p < 0.05, VIP ≥ 1, and FC ≥ 2 or FC ≤ 0.5) were showed in Figure 7A,B. Common differential metabolites for upregulation are 2‐hydroxybutanoic acid and 2‐methoxy‐4‐[(E)‐prop‐1‐enyl]phenol. Common differential metabolites (Table S4) for downregulation are hododeoxycholic acid, glycoursodeoxycholic acid, glycochenodeoxycholic acid, caffeic acid, hippuric acid, hydrocinnamic acid, lysope 14:0, 3‐(3‐hydroxyphenyl)propionate acid, 3‐hydroxyhippuric acid, 2‐hydroxy hippuric acid, 3‐(4‐hydroxyphenyl)‐propionic acid, taurodeoxycholic acid, alpha‐mercholic acid, gamma‐mercholic acid, orthocholic acid, 6‐hydroxy‐3‐succinylpyridine, 7alpha,12beta‐Dihydroxy‐5alpha‐cholan‐24‐oic acid, glycohyodeoxycholic acid, N‐methyltryptamine, 3‐indolepropionic acid, lysopc 14:0, trans‐3‐indoleacrylic acid, and 4‐hydroxyhippurate.

The discovery set and validation set were separately employed for machine learning and subsequent metabolite consistency filtering. The machine learning was conducted using a combination of two modeling approaches, multiML and stepwise, with the intersection of their results as the basis. All findings are detailed in Table S5. Ultimately, a diagnostic panel with optimal performance was derived, comprising nine metabolites: hododeoxycholic acid, 2‐hydroxybutanoic acid, Phe‐Phe, sn‐glycero‐3‐phosphocholine, PysoPE 18:2, PysoPE 16:1, 5‐hydroxyindole‐3‐acetic acid, 2‐(4‐hydroxyphenyl)propionate, and N1‐acetylspermine. The diagnostic efficacy of this panel was assessed using a Cox regression model, as depicted in Figure 7C,D for the discovery and validation sets, respectively. Notably, the panel exhibited robust diagnostic performance, with an AUC of 0.869 for the discovery set and 0.900 for the validation set.

### 3.6 KEGG pathway analysis

**FIGURE 8:**
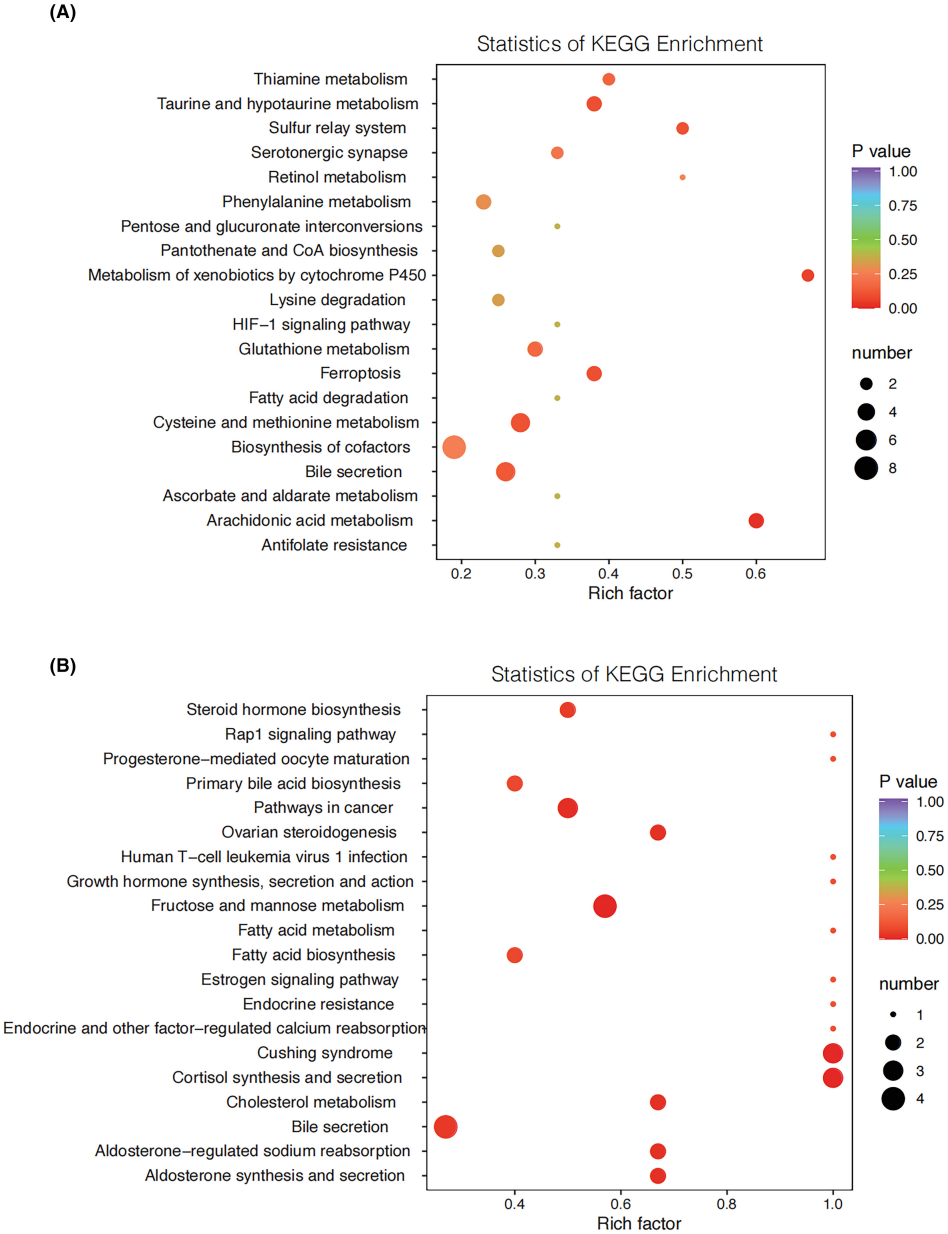
Pathway analysis for discovery set (A) and validation set (B). Based on the differential metabolite results, KEGG pathway enrichment is performed, where Rich factor is the ratio of the number of differentially expressed metabolites in the corresponding pathway to the total number of metabolites detected and annotated by the pathway. The larger the value, the greater the degree of enrichment. The closer the *p*‐value is to 0, the more significant the enrichment is. The size of the points in the figure represents the number of significantly different metabolites enriched in the corresponding pathway.

Following the analysis of differential metabolites, we conducted KEGG pathway enrichment assessments, with the outcomes presented in Figure 8A for the discovery set and Figure 8B for the validation set. The “rich factor” denotes the ratio of differentially expressed metabolites within a given pathway to the total number of annotated metabolites in that pathway. A higher rich factor signifies a more substantial level of enrichment, with greater significance observed as the *p*‐value approaches zero. The size of data points reflects the number of significantly distinct metabolites enriched within each pathway. Notably, a total of 25 pathways exhibited common enrichment in both the discovery and validation sets, with bile secretion being one of the pathways enriched in both datasets.

## 4 DISCUSSION

Malignant tumors are fundamentally genetic diseases^23^ and, concurrently, metabolic diseases.^24^ Their onset, progression, response to radiotherapy and chemotherapy, as well as associated toxic side effects, are all rooted in genetic irregularities, culminating in variations in functional behavior and outcomes. The intricacies of these biochemical processes predominantly emerge as a complex interplay among individual patient factors, antitumor therapies, and the tumor itself. Exploring tumor development and progression through metabolomics offers distinct advantages, including objectivity, precision, efficiency, and a direct perspective. Currently, metabolomics has been employed for the early diagnosis of various cancers, including breast cancer, liver cancer, colorectal cancer, and pancreatic cancer. In this context, it is reasonable to postulate that GCA also possesses its own unique metabolic attributes, which can be harnessed to unveil the underlying physiological and biochemical processes driving this disease. Such insights may even extend to illuminate aberrations in gene regulation.

This study encompassed a substantial cohort of 864 participants, comprising 588 healthy controls and 276 individuals with GCA from two distinct cohorts—the discovery set and the validation set. The primary objective of this investigation was to delineate disparities in serum metabolomics between cardia cancer patients and healthy individuals. The study sought to identify specific serum metabolites associated with GCA, thereby offering insights into the mechanisms underlying disease onset and progression, as well as potential implications for early diagnosis. Furthermore, it aimed to shed light on the responsiveness of cardia cancer to radiotherapy and chemotherapy in subsequent investigations.

A total of 865 detected metabolites were identified, with 25 metabolites consistently found in both the discovery and validation sets. Notably, the majority of these 25 metabolites (comprising 8 bile acids, namely hododeoxycholic acid, glycoursodeoxycholic acid, glycochenodeoxycholic acid, taurodeoxycholic acid, alpha‐mercholic acid, gamma‐mercholic acid, orthocholic acid, and glycohyodeoxycholic acid) were common to both sets. Additionally, the analysis revealed other metabolites, particularly organic acids and their derivatives, including 2‐hydroxybutanoic acid, caffeic acid, hippuric acid, hydrocinnamic acid, and 3‐(3‐hydroxyphenyl)propionate acid, as well as trans‐3‐indoleacrylic acid. Notably, except for 2‐hydroxybutanoic acid, these metabolites exhibited downregulation. Furthermore, the dataset contained three metabolites related to benzene and substituted derivatives. A group of two metabolites common to both sets included amino acids and their derivatives, glycerophospholipids, heterocyclic compounds, organic acids and their derivatives, and tryptamines/cholines/pigments.

Bile acids were initially recognized for their role as digestive aids, facilitating the digestion and absorption of lipids. Ongoing research has expanded our understanding of bile acids, revealing their potential involvement as either carcinogenic agents or cancer suppressors. Indeed, as early as the 1940s, bile acids were implicated as tumor promoters.^25^ In our investigation, we noted a prominent upregulation of bile acids, particularly associated with digestive tract cancers such as throat cancer, esophageal cancer, stomach cancer, pancreatic cancer, and small intestine cancer. However, the specific context of gastric cardia, referred to as Siewert II adenocarcinoma of the esophagogastric junction in Western countries, exhibits a unique anatomical location where the tubular esophagus extends into a sac‐like stomach wall. In this distinct context, we observed a downregulation of bile acids in GCA patients, which stands in contrast to Siewert I and Siewert III tumors, showcasing the unique metabolic profiles of GCA. In summary, our findings indicate that GCA patients possess distinctive metabolic profiles, particularly in the realm of bile acid metabolism. These insights offer a crucial theoretical foundation and innovative strategies for precision‐targeted treatment in GCA patients.

2‐Hydroxybutanoic acid is a significant early detection marker for type 2 diabetes.^26^ Upregulation of 2‐hydroxybutanoic acid serves as a diagnostic and prognostic biomarker for ovarian carcinomas.^27^ The underlying reason for the upregulation of 2‐hydroxybutanoic acid is its involvement in multiple metabolic pathways, one of which is the enhancement of fatty acid oxidation.

No previous studies with larger sample sizes have conducted serum metabolomics analysis in the context of GCA. Nevertheless, a few studies with smaller sample sizes have investigated adenocarcinoma of the esophagogastric junction when compared to healthy controls. Notably, the same upregulated metabolite, hypoxanthine, has been reported in another Chinese cohort.^28^ Hypoxanthine phosphoribosyl transferase 1 (HPRT1) plays a pivotal role in nucleotide recycling, critical for cell growth and biosynthesis, and its upregulation has been observed in various cancers, with implications for clinical outcomes,^29^ including breast cancer^29^ and head and neck squamous cell carcinoma.^30^ Furthermore, we identified common downregulated metabolites in GCA patients, including glycoursodeoxycholic acid and LysoPC(14:0).^31^ Glycoursodeoxycholic acid, a type of bile acid, plays a complex role in carcinogenesis. Traditionally, bile acids have been associated with the development of esophageal cancer, representing one facet of their impact. Conversely, their conjugates have been recognized for their predominantly anticancer properties, suggesting potential opportunities for drug repurposing.^32^ Similar to glycoursodeoxycholic acid, LysoPC(14:0) is a known inducer of lymphocyte and macrophage migration, pro‐inflammatory cytokine production, oxidative stress, and apoptosis, contributing to the exacerbation of inflammation and disease progression.^33^ However, in a contrasting role, it has also demonstrated antitumor effects in lung cancer cells through the regulation of the fatty acid metabolism enzyme, long‐chain acyl‐coenzyme A synthase 5.^34^

Light drinking as well as moderate to heavy alcohol consumption significantly increased the risks of the gastrointestinal cancers than those who remained a non‐drinker in a dose dependent manner, including to esophageal cancer, gastric cancer, and colorectal cancer.^35^ And the main mechanism is likely related to the primary metabolites, acetaldehydes, that have a local toxic effect that increases the risk.^36^, ^37^, ^38^, ^39^ Alcohol dehydrogenase (ADH) and mitochondrial aldehyde dehydrogenase (ALDH) are responsible for metabolizing the bulk of ethanol consumed as part of the diet and their activities contribute to the rate of ethanol elimination from the blood.^40^, ^41^, ^42^ And studies have been confirmed that class IV of ADH isoenzymes significantly higher in gastric cancer tissue than in healthy mucosa.^43^ However, for GCA patients, we have seen nonsignificant distinguish between GCA and healthy control in alcohol and amines metabolites in Figure 6A,B, maybe because of malignancy and sample heterogeneity.

The TNM stage system classifies tumors based on primary tumor characteristics (T), the presence or absence of regional lymph node involvement (N), and the presence or absence of distant metastases.^44^ In our study, advanced stage GCA patients exhibited a significantly worse prognosis compared to those in the early stage (*p*‐values of 0.004 for the discovery set and 0.014 for the validation set). Notably, the heatmap in Figure 6C,D reveals upregulation of amino acids and their metabolites, as well as downregulation of glycerophospholipids in the advanced stage group.

The overexpression of HER2 protein has been demonstrated to instigate malignant transformation in cell culture and transgenic mouse models.^45^ The anti‐HER2 antibody, trastuzumab, has proven to be an effective targeted therapy, showing significant efficacy in the treatment of HER2‐positive breast cancer,^46^ gastric cancer,^47^ and gastroesophageal (GE) junction adenocarcinoma.^48^ Nevertheless, as observed in Figure 5A,B, patients with high HER2 expression exhibited a more favorable prognosis than those in the negative and low‐expression group, although statistical significance was not reached, potentially due to the limited sample size. As a result, we postulate that HER2 may function as a tumor suppressor gene in GCA. Notably, amino acids and their metabolites were upregulated in the HER2 overexpression group, while glycerophospholipids exhibited downregulation in the HER2 negative and low‐expression group.

Metabolomic data were visually represented using heatmaps, with metabolites organized by major classes, as depicted in Figure 6. In summary, the global alterations in the metabolic profile of GCA were markedly distinct when compared to the healthy control group, as shown in Figure 6A,B. These distinctions were particularly prominent in the metabolites related to bile acids, amino acids and their derivatives, glycerolipids, and glycerophospholipids. Furthermore, the differences between early‐stage and advanced stage GCA patients were evident in Figure 6C,D. Amino acids and their metabolites exhibited upregulation, while glycerophospholipids were downregulated. Finally, disparities in HER2 expression between the negative and low‐expression groups and the high expression group are evident in Figure 6E,F. Notably, amino acids and their metabolites showed upregulation in the HER2 overexpression group, while glycerophospholipids exhibited downregulation in the HER2‐negative and low‐expression group.

We have established a diagnostic panel for GCA, which encompasses the following metabolites: hododeoxycholic acid, 2‐hydroxybutanoic acid, Phe‐Phe, sn‐glycero‐3‐phosphocholine, PysoPE 18:2, PysoPE 16:1, 5‐hydroxyindole‐3‐acetic acid, 2‐(4‐hydroxyphenyl) propionate, and N1‐acetylspermine. The results of the Cox regression model for this panel are illustrated in Figure 7C,D, corresponding to the discovery set and the validation set, respectively. These findings attest to the panel's robust diagnostic efficacy.

Bile secretion exhibited enrichment in both the discovery set and the validation set. The intake of saturated fats triggers elevated bile secretion into the intestine. This increased bile secretion favors the growth of digestive tract microorganisms with the capacity to modify the bile acid composition, leading to the production of tumorigenic secondary bile acids, such as deoxycholic acid and lithocholic acid.^49^

## AUTHOR CONTRIBUTIONS

**Li Dong Wang:** Conceptualization (lead); funding acquisition (supporting); supervision (equal); writing – review and editing (equal). **Meng Xia Wei:** Conceptualization (equal); data curation (equal); formal analysis (equal); funding acquisition (equal); investigation (equal); methodology (equal); project administration (equal); resources (equal); software (equal); supervision (equal); validation (equal); visualization (equal); writing – original draft (equal); writing – review and editing (equal). **Zheng Yang:** Software (equal); writing – review and editing (equal). **Pan Pan Wang:** Resources (equal); software (equal); writing – review and editing (equal). **Xue Ke Zhao:** Writing – review and editing (equal). **Xin Song:** Writing – review and editing (equal). **Rui Hua Xu:** Writing – review and editing (equal). **Jing Feng Hu:** Writing – review and editing (equal). **Kan Zhong:** Writing – review and editing (equal). **Ling Ling Lei:** Writing – review and editing (equal). **Wen Li Han:** Writing – review and editing (equal). **Miao Miao Yang:** Writing – review and editing (equal). **Fu You Zhou:** Writing – review and editing (equal). **Xue Na Han:** Writing – review and editing (equal). **Zong Min Fan:** Writing – review and editing (equal). **Jia Li:** Supervision (equal); validation (equal); writing – review and editing (equal). **Ran Wang:** Investigation (equal). **Bei Li:** Investigation (equal).

## FUNDING INFORMATION

This study was funded by the National Natural Science Foundation of China (Grant number: 81872032) and 2021 Henan Medical Science and Technology Research Plan Joint Construction Project (LHGJ20210336).

## CONFLICT OF INTEREST STATEMENT

The authors disclose no conflicts.

## Supporting information


Table S1.

## Data Availability

All relevant data are within the manuscript and its Additional files. And if you have any questions and reasonable request please contact corresponding author (ldwang2007@126.com).
